# Supplementary material for: Knockout of AMPA receptor binding protein Neuron-specific gene 2 (NSG2) enhances associative learning and cognitive flexibility
Source: Mol Brain. 2024 Dec 18;17:95. doi: 10.1186/s13041-024-01158-7 (PMC11654403; doi:10.1186/s13041-024-01158-7)
Supplement: Supplementary file 1 — Supplementary Material 1 [file 13041_2024_1158_MOESM1_ESM.docx]

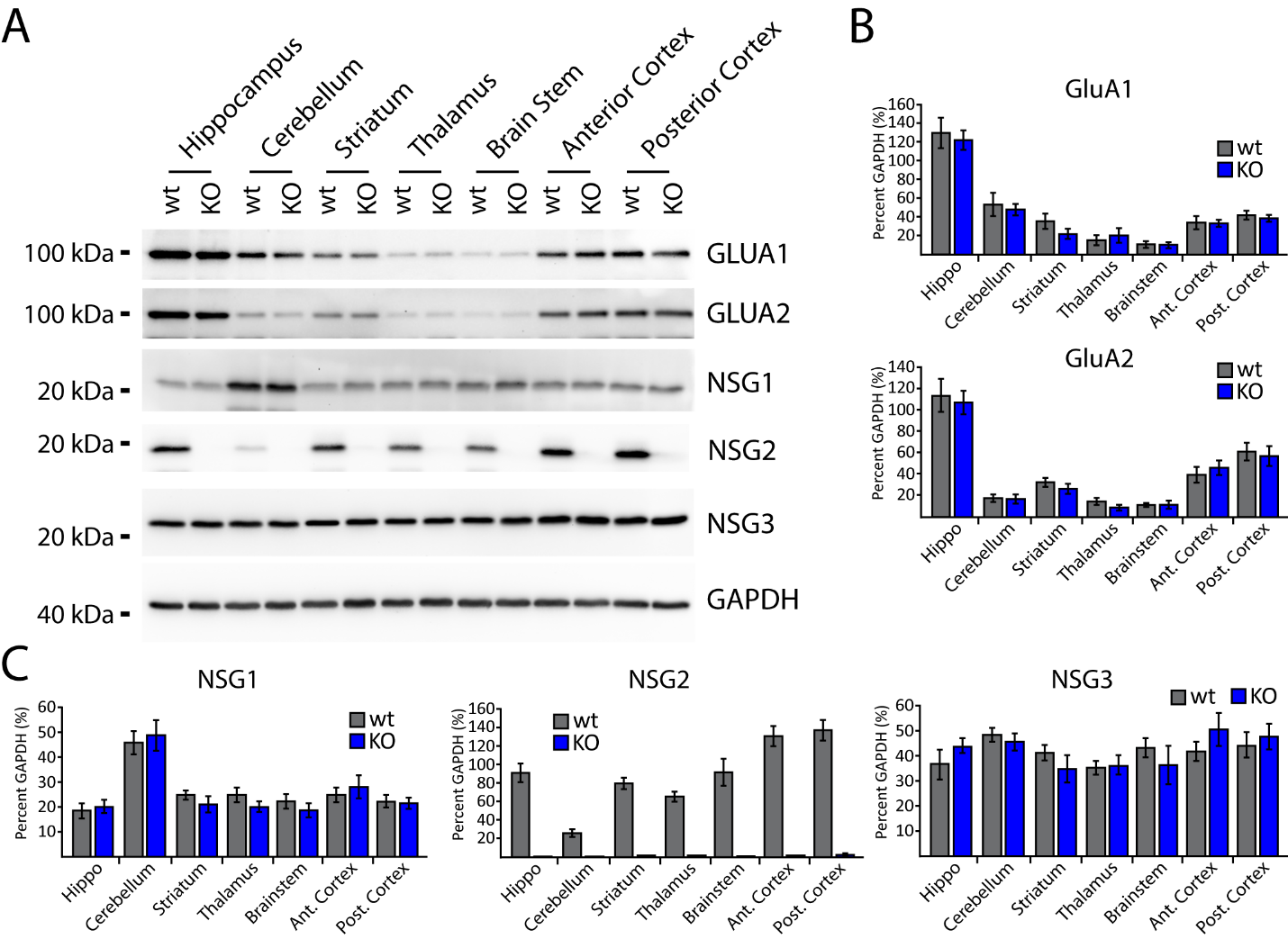


**Supplementary Figure 1.** Related to Figure 1. (A) Representative Western blots of microdissected brain regions from a wild-type and NSG2 KO animal that were probed for various proteins. (B) Pooled data from multiple blots (n=3 biological replicates) demonstrate a significant main effect of brain region for GAPDH-normalized GLUA1 (F_(6, 28)_= 52.32; p<0.001) and GLUA2 (F_(6, 28)_= 55.97; p<0.001) expression. No significant effect of genotype was observed for either GAPDH-normalized GLUA1 (F_(1, 28)_= 1.39; p=0.25) or GLUA2 (F_(1, 28)_= 1.6; p=0.69). (C) Pooled data from multiple blots (n=3 biological replicates) demonstrate a significant main effect of brain region on GAPDH-normalized NSG1 (F_(6, 28)_= 9.03; p<0.001), NSG2 (F_(6, 28)_= 6.15; p<0.001) and NSG3 (F_(6, 28)_= 3.01; p=0.02) expression. A significant effect of genotype was observed for GAPDH-normalized NSG2 (F_(1, 28)_= 170.8; p<0.001), but no effect of genotype was observed for either NSG1 (F_(1, 28)_= 0.14; p=0.71) or NSG3 (F_(1, 28)_= 0.08; p=0.77). Data are presented as mean and SEM.


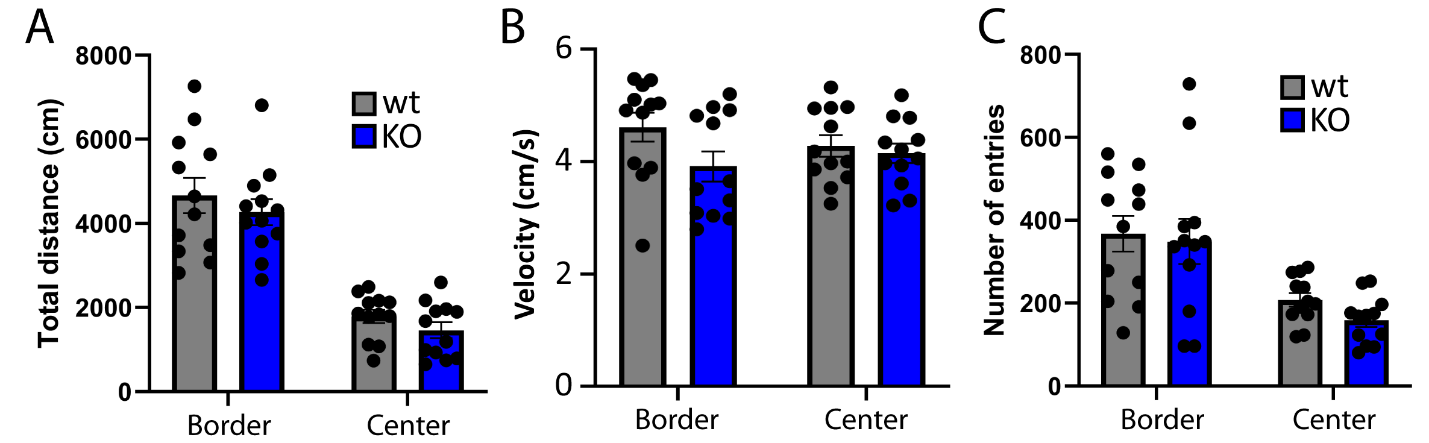


**Supplementary Figure 2.** Related to Figure 3. NSG2 KO animals do not differ from wild-type animals on Open Field task measures. (A) Number of entries into the Border or Center areas of the Open Field box. (B) Velocity in each area of the Open Field box. (C) Distance traveled in each area of the Open Field box. Data are presented as mean and SEM with individual animals represented by each dot.


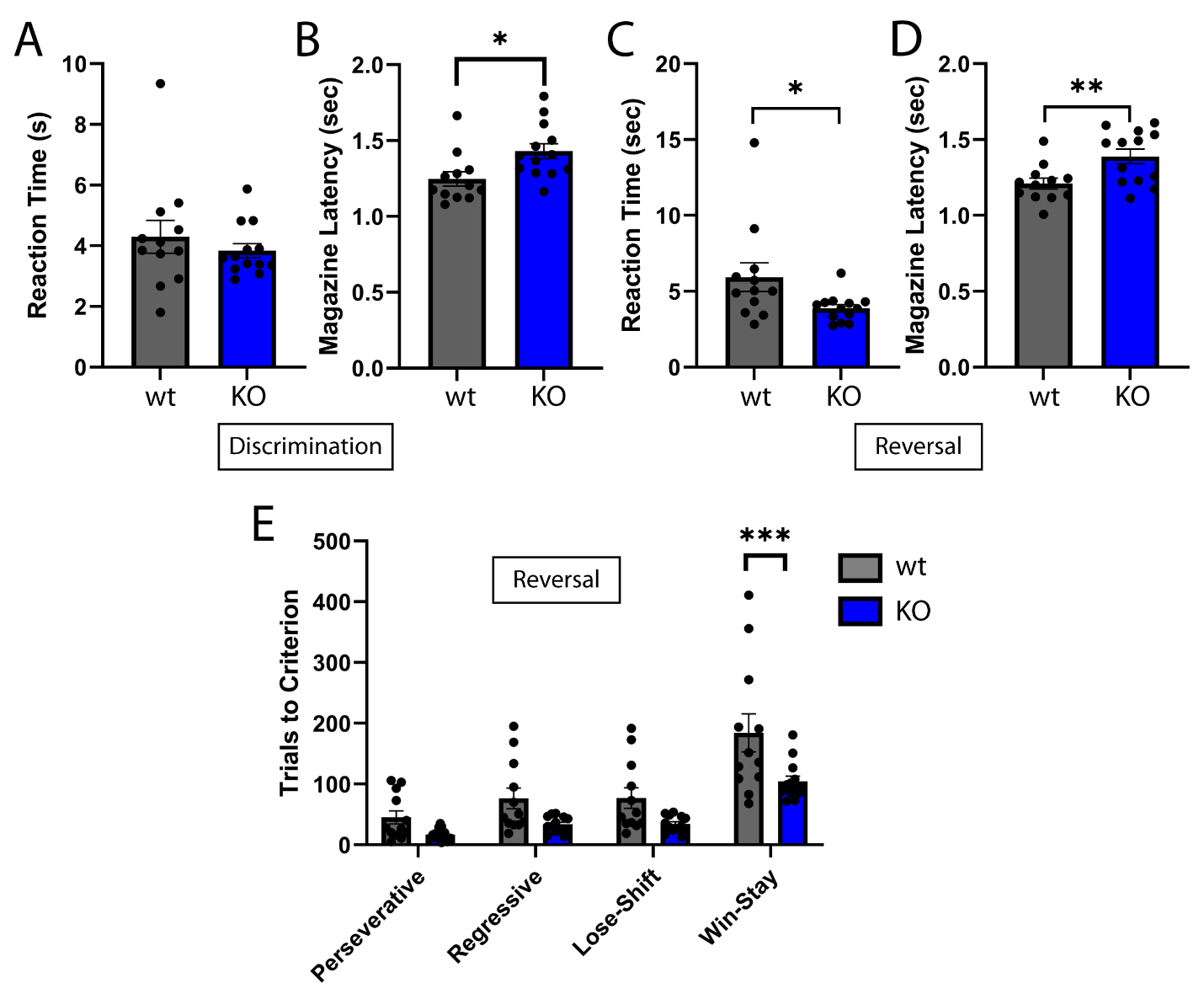


**Supplementary Figure 3.** Related to Figure 5. NSG2 KO animals display significant differences in multiple measures during touch-screen Discrimination-Reversal testing. Reaction time (A) and magazine latency (B) during the initial Discrimination on the touchscreen D-R task. Reaction time (C) and magazine latency (D) during Reversal on the touchscreen D-R task. E. Average number of trials for each sub-trial category including perseverative, regressive, lose-shift and win-stay during the late (>50% correct) Reversal stage of the touchscreen D-R task. Data are presented as mean and SEM with individual animals represented by each dot. *p-value <0.05, **<0.01 by two-tailed independent t-test. In E, ***p-value <0.001 represents Bonferroni’s multiple comparisons following 2-way ANOVA.


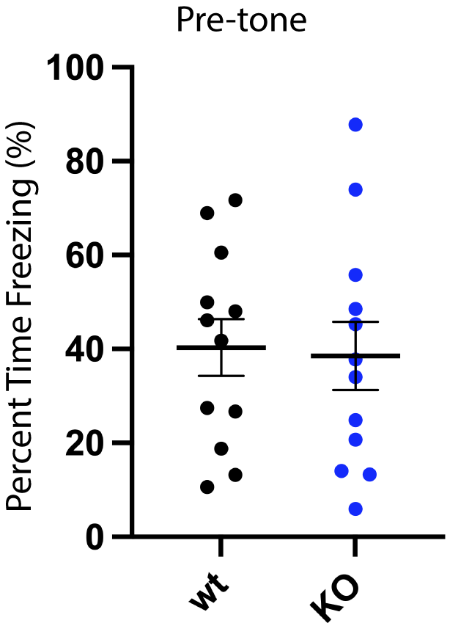


**Supplementary Figure 4.** Related to Figure 7. NSG2 animals spent equivalent amounts of time freezing prior to tone delivery on day 2 testing. Distribution of pre-tone freezing times was not significantly different between groups (t_(22)_ = 0.1904, *P* = 0.85).


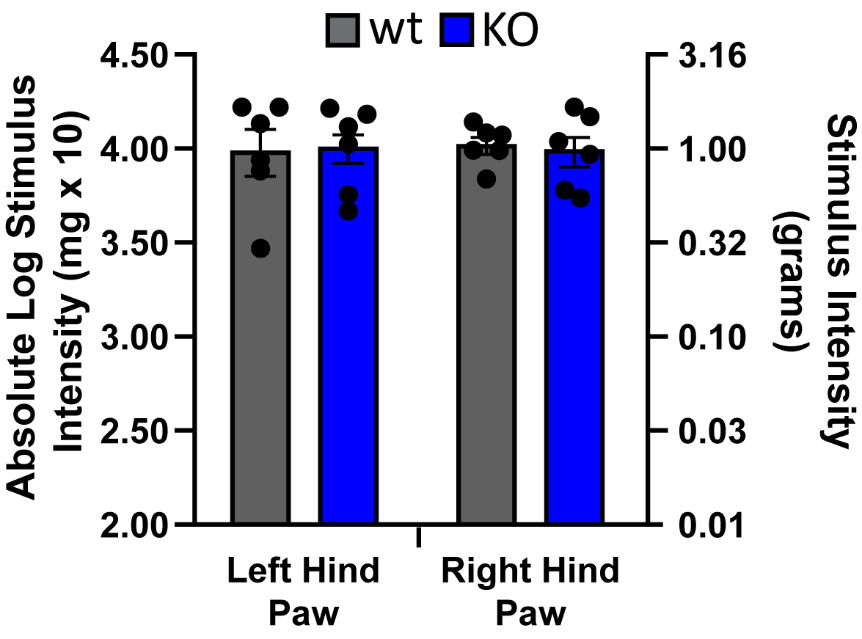


**Supplementary Figure 5.** Related to Figure 7. NSG2 KO animals do not show enhanced somatosensation. Bar graphs represent mean mechanical intensity necessary to elicit hindpaw withdrawal via application of Von Frey hairs. Wild-type and NSG2 KO animals did not show significant differences in sensitivity in either left (p=0.88) or right (p=0.69) hindpaws (n=6/group).
